# Distinction of non-specific low back pain patients with proprioceptive disorders from healthy individuals by linear discriminant analysis

**DOI:** 10.3389/fbioe.2022.1078805

**Published:** 2022-12-13

**Authors:** Seyed Mohammadreza Shokouhyan, Mehrdad Davoudi, Maryam Hoviattalab, Mohsen Abedi, Soha Bervis, Mohamad Parnianpour, Simon Brumagne, Kinda Khalaf

**Affiliations:** ^1^ Department of Mechanical Engineering, Sharif University of Technology, Tehran, Iran; ^2^ Clinic for Orthopaedics and Trauma Surgery, Heidelberg University Hospital, Heidelberg, Germany; ^3^ Department of Physiotherapy, Faculty of Rehabilitation, Shahid Beheshti University of Medical Sciences, Tehran, Iran; ^4^ Physical Therapy Department, School of Rehabilitation Sciences, Shiraz University of Medical Sciences, Shiraz, Iran; ^5^ Rehabilitation Sciences Research Center, Shiraz University of Medical Sciences, Shiraz, Iran; ^6^ Department of Rehabilitation Sciences, KU Leuven, Leuven, Belgium; ^7^ Department of Biomedical Engineering, Healthcare Engineering Innovation Center, Khalifa University of Science and Technology, Abu Dhabi, United Arab Emirates

**Keywords:** non-specific low back pain, linear discriminant analysis, COP, posture control, proprioceptive impairment

## Abstract

The central nervous system (CNS) dynamically employs a sophisticated weighting strategy of sensory input, including vision, vestibular and proprioception signals, towards attaining optimal postural control during different conditions. Non-specific low back pain (NSLBP) patients frequently demonstrate postural control deficiencies which are generally attributed to challenges in proprioceptive reweighting, where they often rely on an ankle strategy regardless of postural conditions. Such impairment could lead to potential loss of balance, increased risk of falling, and Low back pain recurrence. In this study, linear and non-linear indicators were extracted from center-of-pressure (COP) and trunk sagittal angle data based on 4 conditions of vibration positioning (vibration on the back, ankle, none or both), 2 surface conditions (foam or rigid), and 2 different groups (healthy and non-specific low back pain patients). Linear discriminant analysis (LDA) was performed on linear and non-linear indicators to identify the best sensory condition towards accurate distinction of non-specific low back pain patients from healthy controls. Two indicators: Phase Plane Portrait _ML_ and Entropy _ML_ with foam surface condition and both ankle and back vibration on, were able to completely differentiate the non-specific low back pain groups. The proposed methodology can help clinicians quantitatively assess the sensory status of non-specific low back pain patients at the initial phase of diagnosis and throughout treatment. Although the results demonstrated the potential effectiveness of our approach in Low back pain patient distinction, a larger and more diverse population is required for comprehensive validation.

## 1 Introduction

Likely driven by ageing and population increase, the Global Burden of disease Report reveals a significant increase in both the number of Low back pain (LBP) patients and the prevalence of LBP in all age groups worldwide in the last 2 decades (1990–2019). The same report also points out that although the prevalence of LBP increases with increasing age, currently, the 50–54 years age group has the highest LBP prevalence (Vos et al., 2021). Expectedly, such trend in an active population is associated with grave health and economic consequences. (Airaksinen et al., 2006; Balagué et al., 2012). Globally, LBP continues to be the main cause of years lived with disability or YLDs. Health economists have estimated that the caring costs for 15% of people with low back pain are equivalent to 85% of those associated with the remaining population (Hashemi et al., 1997; Hashemi et al., 1998; Filiz et al., 2005). In the US alone, health economists estimate that up to 624 billion dollars are spent annually on direct and indirect expenses associated with LBP (Dagenais et al., 2008). Importantly, it has been shown that there is no particular identifiable pathoanatomical etiology for LBP in 85–95 percent of patients who visit primary care doctors, hence the common diagnosis of non-specific low back pain (NSLBP) (Airaksinen et al., 2006; Hancock et al., 2011). The exact mechanisms and causes of NSLBP, which remain elusive, are therefore highly relevant in low back pain investigations (Costa et al., 2013).

Optimal postural control is task-dependent and dynamically variable during the performance of different daily activities. The human brain performs diverse targeted control adjustments on the ankle, knee, hip and spine in order to maintain postural stability (Allum et al., 1998). The central nervous system (CNS), in turn, adapts and selects the appropriate sensory input signals according to changes in postural conditions. The CNS, subsequently, reweights (dynamically adjusts the weight assigned to a particular signal) visual, vestibular, and proprioceptive input to generate the appropriate muscle forces required for the particular task. This allows effective control of the center of mass, resulting in proper equilibrium of the body (Brumagne et al., 2004; Carver et al., 2006). Previous investigations explored different models and methods for preserving equilibrium (Horak and Nashner, 1986; Runge et al., 1999). Several hypothesized postural control strategies can be interpreted by the infamous inverted pendulum model. The ankle strategy suggests that stability is maintained by balancing the body initially around the ankle joint (Horak and Nashner, 1986). This strategy is adequate for simple conditions, such as standing on a rigid surface, but is less successful in more complex conditions, including foam and other unstable surfaces. According to the inverted model theory, sufficient motion has to be generated at the trunk and the hip joint (hip strategy) for optimal postural control during more complex conditions (Horak and Nashner, 1986). On the other hand, multi segmental postural control models delineate that postural control is provided not only by specific modifications at one joint, but also include various modifications at different joints performed by the CNS (Morasso and Schieppati, 1999; Schieppati et al., 2002; Kiemel et al., 2008).

The literature demonstrates controversy regarding the causal association between postural control and LBP. Several studies concluded that postural control Impairment can be considered as one of the causal factors associated with non-specific low back pain (NSLBP) (Peterka, 2002; Horak, 2006; Ruhe et al., 2011), while others maintained that that there is no consensus regarding the link between posture and low back pain (Swain et al., 2020). Although NSLBP Patients have demonstrated impaired postural control during complicated conditions (standing on unstable support surfaces, eyes closed, etc.), only a few prospective investigations looked into the cause-and-effect relationship between the development of LBP and impaired proprioceptive postural control. One study demonstrated that improper lumbar repositioning and increased posterior pelvic tilt while sitting enhance the likelihood of developing LBP in nursing students (Mitchell et al., 2010). Another study indicated that LBP in college athletes was exacerbated by delayed trunk muscular responses during sitting (Cholewicki et al., 2005). Proprioceptive impairment was implicated as the underlying mechanism in both prospective studies, however direct assessment of the proprioceptive system was missing from both investigations. Due to the lack of evaluation and agreement on the association between posture and low back pain, no clear causation can be construed regarding the role of postural control impairment in the development of LBP. On the other hand, it has been demonstrated that compared to healthy individuals, NSLBP patients exhibit altered proprioceptive postural control, in which the patients adopt a body and trunk stiffening strategy and rely more on ankle proprioception to control their posture during quiet upright standing (Brumagne et al., 2008). This highlights the potential of using the different sensory reweighting strategy as means of distinguishing NSLBP patients from healthy individuals.

Many previous studies focused on the motor output of postural control in NSLBP patients (Ruhe et al., 2011). Others advocated the importance of considering the sensory inputs, particularly in terms of weighting the various proprioceptive signals, a critical consideration in optimal postural control (Gandevia et al., 1992; Lackner and DiZio, 2005; Carver et al., 2006). It is indeed well established that musculoskeletal injuries (Haghighat et al., 2021a; Haghighat et al., 2021b; Haghighat et al., 2021c; Haghighat et al., 2021d; Davoudi et al., 2022a), neuromusculoskeletal disease, such as stroke, Parkinson’s, Multiple Sclerosis, Chronic obstructive pulmonary disease (Davoudi et al., 2022b) and other psychological factors including anxiety, are associated with disrupting the function of the sensory systems (Jamali et al., 2019; Dehmiyani et al., 2022). Change in sensory input function has also been observed among patients with NSLBP (Newcomer et al., 2000). These changes, typically impact several physiological functions including reduced sensory acuity (Sharma and Pai, 1997), altered muscle recruitment patterns (van Dieën et al., 2003; Hodges et al., 2009), and reorganization of the somatosensory regions of the brain cortex (Flor et al., 1997). Peripheral simulation of muscle spindles has revealed that LBP patients rely on their ankle muscle proprioception more than the optimal weighted ankle and back muscle proprioception strategy common in people free of back pain (Brumagne et al., 2008; Claeys et al., 2011). It is believed that in this patient population, the CNS increases ankle proprioception gain, reflecting impaired proprioceptive weighing capacity. Indeed, in challenging postural circumstances, such as standing on unstable surfaces, when the ankle strategy is no longer an appropriate option for the CNS, less reliable postural control is detected in LBP patients (Kiers et al., 2012), and sensory input integration is less optimal due to the decrease in proprioceptive signals from back muscles leading to impaired postural control (della Volpe et al., 2006; Brumagne et al., 2008; Claeys et al., 2011; Mok et al., 2011). Abnormal spinal loading, pain and recurrence of NSLBP are believed to result from such decrease in the proprioceptive function and the consequent impairment of postural control (Claeys, 2013).

In the past several decades, many relevant studies investigated the biomechanics of the low back by using various approaches, tools and technologies to distinguish NSLBP patients from healthy individuals. Marras et al., (1993) and Marras et al., (1995) found significant differences in angular velocity and acceleration discriminating low back pain patients from healthy controls by using a custom-designed three axial goniometer. Classification techniques were also utilized based on coefficients assigned to individuals in eleven subgroups (patients with 10 different levels of low back pain and one healthy cohort) to quantify the presence and intensity of LBP. The combined spinal movement in LBP patients, measured with a three dimensional electromagnetic tracking system, demonstrated reduced movement as compared with healthy individuals (Barrett et al., 1999). Some research groups used Ultrasonic measurement systems (Vogt et al., 2001) to study the impact of non-specific low back pain on 3-D kinematics, while others focused on dynamic pelvic and thoracic oscillations, which were observed to increase as a function of NSLBP. Several studies (Ferreira et al., 2004; van der Hulst et al., 2010; Bervis et al., 2020) focused on abdominal and lumbar muscle activity in chronic low back pain patients, demonstrating increased muscle activity of the erector spinae and rectus abdominis in CLBP patients during walking and moving a flexi bar. Laird et al. (2014) measured the lumbo-pelvic movement in people with and without low back pain, demonstrating that compared to those without LBP, LBP patients showed decreased lumbar ROM, decreased proprioception, and moved more slowly. Scholtes et al. (2009) investigated the lumbopelvic movement in LBP patients and healthy control cohorts measured by a three dimensional motion capture system. They discovered that those with low back pain had an earlier lumbopelvic rotation and a larger maximum lumbopelvic rotation angle compared to healthy individuals during knee flexion and hip lateral rotation. Ashouri et al. (2017) analyzed trunk motion of LBP and healthy controls during several tasks measured by IMU sensors. A Support Vector Machine classifier methodology was utilized for data classification, which enabled the distinction between the two groups with high accuracy and sensitivity. More recently, smart wearables and sensor-based methodologies have yielded encouraging results for the quantification and classification of LBP (Davoudi et al., 2020). These novel technologies, along with big data management tools (AI, ML, SVM, etc.) promise a paradigm shift towards low-cost portable continuous monitoring personalized precision medicine.

Although the literature, including the above-mentioned studies, is rich with quantitative investigations exploring the various dimensions of NSLBP, the multifactorial etiology of the disease and its negative impact on today’s world remain unresolved challenges. Elusive issues include understanding the role of motor control in NSLBP patients and the distinctive motor control strategies adopted by these patients for preserving postural stability. To augment the findings in literature, this investigation was designed to scrutinize the best sensory condition to distinguish NSLBP patients from healthy controls leveraging different surface, and vibration conditions, as well as, linear and non-linear mathematical tools. We hypothesize that the sensory condition of standing on a foam surface with induced vibration, in conjunction with non-linear mathematical tools, can provide a suitable framework to differentiate LBP patients from healthy individuals.

## 2 Materials and methods

### 2.1 Ethics statement

The University Internal Ethics Board evaluated and authorized the study involving human subjects (IRB Shahid Beheshti University of Medical Sciences, No: IR.SBMU.RETECH.REC.1396.1392). Prior to beginning the trial, participants were fully informed and given their consent.

### 2.2 Participants

The experimental set up in this study is similar to our prior investigation on chronic low back pain patients (Shokouhyan et al., 2020) including male participants who suffered LBP at least three times in the recent year or one time in a 3 months duration. The participants were divided evenly between the two groups: an NSLBP group and a healthy control group based on two questionnaires. The ODI (Oswestry Disability Index) (Fairbank and Pynsent, 2000) and the NPRS (quantification of pain) questionnaires were completed by each individual for the assessment of LBP and to rate the pain on a numerical scale, respectively (Joos et al., 1991). Patients who reported an ODI >6 or NPRS >0 were categorized as part of the “NSLBP” group. In this study, however, all males in the “healthy” group reported zero on both the NPRS and ODI questionnaires. The experimental protocol dictated that the experiment would be postponed if any participant reported pain, although none of participants reported any pain during the current experimental tests. The NSLBP patients had to be free of any neurological, respiratory, or cardiovascular disease, as well as any spinal, neck, chest, or lumbar surgeries, in order to meet the inclusion criteria. 5 subjects were excluded due to not meeting the inclusion criteria and the experimental tests were performed on 20 Chronic Non-Specific Low Back Pain (CNSLBP) patients. Demographic data, including age, height, weight, and BMI index are presented in Table 1.

**TABLE 1:** Participants’ demographic information for both healthy and low back patients.

Variables	Healthy	CNSLBP	*p*-value
N (Gender)	20 (Male)	20 (Male)	
Age	$25.5\left(\pm 0.7\right)$	$24.5\left(\pm 0.9\right)$	0.2
Height (cm)	$174\left(\pm 6.5\right)$	$172\left(\pm 7.5\right)$	0.316
Weight (kg)	$64\left(\pm 8.6\right)$	$62\left(\pm 7.5\right)$	0.26
BMI kgm2	$20.3\left(\pm 2.3\right)$	$21.7\left(\pm 2.4\right)$	0.97

### 2.3 Muscle proprioception

According to prior investigations, muscle vibration is the most common method for altering proprioception inputs (Goodwin et al., 1972; Roll and Vedel, 1982). In order to bias the soleus and lumbar muscles’ proprioception in this investigation, we developed an in-house vibrating apparatus with four brushless DC motors. The triceps surae, which is located in the calf of the lower legs, and the longissimus and multifidus muscles, which span the lumbar vertebrae L3 to L5, were also targeted for placement of the device. Based on previous studies, the vibration frequency of our device was adjusted to 70 Hz, with an amplitude of around 0.5 mm, to provide the best altered proprioceptive data (Goodwin et al., 1972; Roll and Vedel, 1982; Cordo and Gurfinkel, 2004).

### 2.4 Procedure

A force plate was used for collecting the center-of-pressure (COP) body fluctuation data for each subject (Bertec, Columbus, OH, United States). The kinematic data was captured using a Vicon optical motion capture system with reflective markers synced to the force plate. According to the literature, the markers were attached to the C7, T12, lower sternum (xiphoid process), clavicle (Incisura jugularis), right scapula, right and left sides of the PSIS (posterior superior iliac spine), and ASIS (anterior superior iliac spine). On both devices, the sampling frequency was set to 100 Hz. The motor straps were fastened to the bilateral multifidus muscles and the muscular spindle of the triceps surae on each leg. Each subject completed 8 randomized different trials while having their eyesight obscured (by wearing an eye mask) as follows: 1) standing on a motionless rigid surface (without any vibrator-induced movement); 2) standing on a rigid surface with the activation of the triceps vibrators; 3) standing on a rigid surface with the activation of the multifidus vibrators; 4) standing on a rigid surface with the activation of both the triceps and multifidus vibrators; 5) standing on a motionless foam surface; 6) standing on a foam surface with the activation of the triceps vibrators; 7) standing on a foam surface with the activation of the multifidus vibrators; and 8) standing on a foam surface with the activation of both the triceps and multifidus vibrators. The trunk angles were recorded in the three anatomical planes for each trial, and the COP data was obtained in the anterior posterior (AP) and medial lateral (ML) directions. Each trial lasted 30 s: for the first 10 s of each trial, the subject stood on the force platform without experiencing any vibration (the balancing phase); for the next 20 s, the motors were turned on at a frequency of 70 Hz (the vibration phase).

### 2.5 Linear indicators of COP time series

According to the literature, a second-order Butterworth non-linear filter was used to filter the data (Ghomashchi et al., 2011) using a 5 Hz cutoff frequency. The mean total velocity, the standard deviation of displacement, and the phase plane portrait for both anterior-posterior (AP) and medial-lateral (ML) directions were obtained in accordance with Table 2 in order to analyze the center-of-pressure data. $\bar{x}$ is the average of the balance time series, $x_{i}$ represents each point of the vibration time series, and N denotes the length of the time series.

**TABLE 2:** Linear parameters of COP analysis (Salavati et al., 2009).

Parameter	Formula
SD of Amplitude (mm)	
AP Eq. 1	$\sigma_{x}=\sqrt{\frac{\sum {\left(x_{i}-\bar{x}\right)}^{2}}{N-1}}$
ML (Eq. 2)	$\sigma_{y}=\sqrt{\frac{\sum {\left(y_{i}-\bar{y}\right)}^{2}}{N-1}}$
SD of Amplitude ( mm/s )	
AP (Eq. 3)	$\sigma_{v_{x}}=\sqrt{\frac{\sum {\left(v_{x_{i}}-\bar{v}\right)}^{2}}{N-1}}$
	$v_{x_{i}}=\frac{x_{\left(i+1\right)}-x_{i}}{t_{\left(i+1\right)}-t_{i}}$
ML (Eq. 4)	$\sigma_{y}=\sqrt{\frac{\sum {\left(v_{y_{i}}-\bar{v}\right)}^{2}}{N-1}}$
	$v_{y_{i}}=\frac{y_{\left(i+1\right)}-y_{i}}{t_{\left(i+1\right)}-t_{i}}$
Mean total velocity ( mm/s )	
(Eq. 5)	$\bar{V}=\frac{1}{T}\overset{T}{\underset{i=1}{\sum }}\sqrt{{\left(x_{i+1}-x_{i}\right)}^{2}+{\left(y_{i+1}-y_{i}\right)}^{2}}$
Phase plane portrait (Arbitrary unit)	
AP (Eq. 6)	$\sigma_{rx}=\sqrt{{\sigma_{x}}^{2}+{\sigma_{v_{x}}}^{2}}$
ML (Eq. 7)	$\sigma_{ry}=\sqrt{{\sigma_{y}}^{2}+{\sigma_{v_{y}}}^{2}}$
AP-ML (Eq. 8)	$\sigma_{r}=\sqrt{{\sigma_{rx}}^{2}+{\sigma_{ry}}^{2}}$

Unit of measures are as follow: $\sigma_{x}$ and $\sigma_{y}$ are in mm, $\sigma_{v_{x}}$ , $\sigma_{v_{y}}$ and $\bar{V}$ are in mms , $\sigma_{r_{x}}$ , $\sigma_{r_{y}}$ and $\sigma_{r}$ are in arbitary unit.

### 2.6 RQA and Lyapunov non-linear indicators of COP time series and trunk sagittal angle

The phase space of the COP and trunk sagittal angle data was reconstructed in MATLAB (MathWorks, Inc., Natick, Massachusetts, United States) using average Mutual Information (AMI) and False Nearest Neighbors (FNN) methods, which are the conventional techniques for finding the time-delay parameter and the embedding dimension parameter, respectively (Horak et al., 2003). The AP and ML directions of the COP, and trunk sagittal angle were the three signals that were used to rebuild the phase space for each subject. The COP and trunk angle’s space embedding dimensions were typically 3 in most cases. The first minimum relative for each participant was considered as the time delay.

By using Recurrence Quantification Analysis (RQA) approach, the features of complexity and amount of recurrence can be calculated as dynamic properties of an observed time series. Riley et al. (1999) extracted numerical criteria based on diagonal lines in recurrence plot (RP). In this study, RQA quantitative measurements were computed by the RQA software (Webber Jr, 2009), developed by Webber Jr and Zbilut (2005). According to Riley et al. (1999), the neighborhood radius was calculated as 2.5 percent of the mean distance using the Euclidean norm.

Upon phase space reconstruction for the COP and trunk angle time series, short and long terms Lyapunov were calculated as stability indicators (more negative indicates more stable) of the dynamic path in reconstructed phase domain according to Rosenstein et al. (1993). Based on the initial slope of the curve during the first few sample intervals, the short-term time ( $\lambda_{S}$ ) scale was obtained. Similarly, the slope of the function following the rising interval was used to get the long-term Lyapunov ( $\lambda_{L}$ ) exponent.

### 2.7 Linear discriminant analysis

**FIGURE 1:**
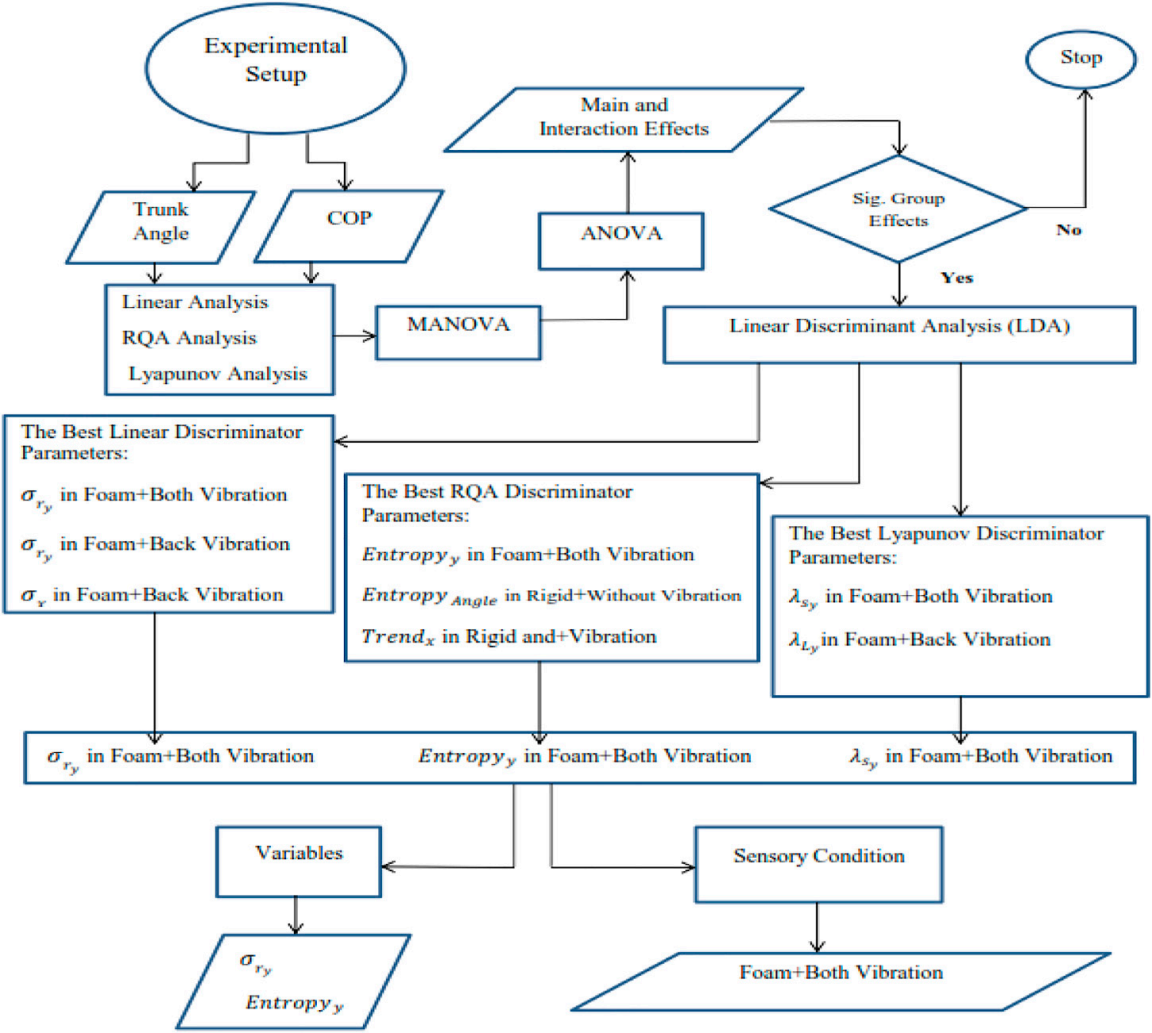
Flow chart of statistical analysis.

Normal distribution was verified using a MATLAB Lillie test ((MathWorks, Inc., Natick, MA, United States) for all the calculated parameters (COP and trunk angle for 2 groups, 4 vibration areas and 2 placement conditions). All the parameters were normally distributed, and hence, analysis of variance (ANOVA) and multiple analysis of variance (MANOVA) tests were used to compare linear and non-linear indicators of the COP and trunk data in order to investigate statistically significant differences. The group (healthy or NSLBP), vibration location (triceps, multifidus, none, and both), and foot positioning condition were taken into consideration as the independent variables in this study (rigid or foam) resulting in a 2×4×2 experimental design configuration. At a level of p<0.05 , the results were considered significant (Field, 2013). A scheme was developed (Figure 1) to identify the best sensory conditions and performance parameters to separate the groups. Linear Discriminant Analysis (LDA) was performed on linear, RQA and Lyapunov parameters separately. Representatives of each analysis variable group were compared with each other, and the best dependent variables with the best sensory condition were determined. This procedure can be seen in Figure 1.

## 3 Results

**FIGURE 2:**
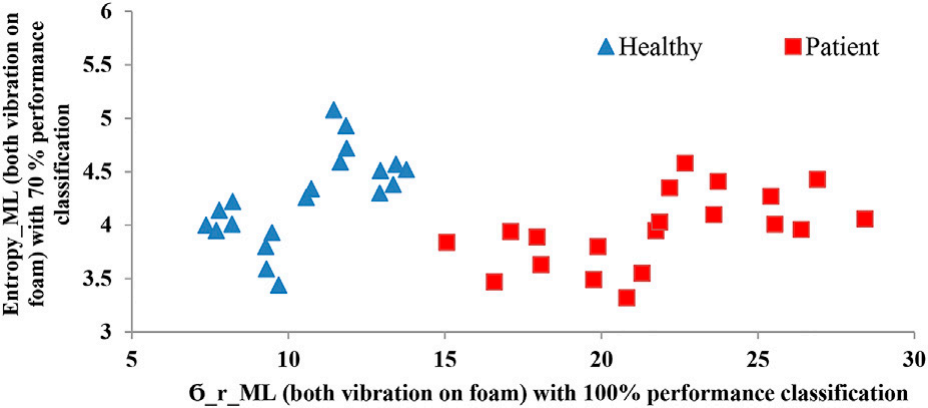
Results of the discriminant analysis.

The results of the ODI and NPRS questionnaires demonstrate significant differences between the healthy participants and the NSLBP group, as shown in Table 3. The ANOVA results are depicted in Supplementary Tables SA1–SA3. The linear discriminant analysis demonstrated that $\sigma_{r_{y}}$ and Entropy ML were the best parameters to distinguish NSLBP and Healthy groups in this study, with a performance classification percentage of 100% and 70%, respectively, while the foam surface in conjunction with vibration presented the best sensory condition (Figure 2). As shown in Figure 2, NSLBP and Healthy individuals of this study can be successfully discriminated with these two parameters. The two groups can be separated by a line, although the line formula is not clear due to a possible overlap in a bigger membership group.

**TABLE 3:** Oswestry disabity inventory questionnaire and pain scale results from participants.

Questioners	Healthy (SD)	Patient (SD)	Significant difference
ODI-2 (0–100)	0	12.3 (3.6)	Yes
NPRS (0–10)	0	2.5 (1.2)	Yes

The correlation Matrix of the significant relationship amongst the eight chosen performance parameters and selected primary condition are indicated in Table 4. It is not surprising that many of these candidates are highly correlated. As the table reveals, the final model that completely separated the two groups kept one measure from the linear and one from the RQA analyses representatives ( $\sigma_{r_{y}}$ and Entropy ML), in conjunction with the foam surface and vibration of both the ankle and back.

**TABLE 4:** Correlation matrix for LDA analysis representatives.

Discriminant parameters	$\sigma_{r_{y-8}}$	$\sigma_{r_{y-7}}$	$\sigma_{x-7}$	${Entropy}_{\;y-8}$	${Entropy}_{\;Angle-1}$	${Trend}_{x-3}$	$\lambda_{s_{y-8}}$	$\lambda_{L_{y-7}}$
$\sigma_{r_{y-8}}$	**1**	**0.94** ^a^	**0.77** ^a^	**-0.1**	**0.47** ^a^	**-0.71** ^a^	**0.53** ^a^	0.6^a^
$\sigma_{r_{y-7}}$		**1**	**0.63** ^a^	**-0.11**	**0.42** ^a^	**-0.64** ^a^	**0.41** ^a^	0.65^a^
$\sigma_{x-7}$			**1**	**0.3**	**0.57** ^a^	**-0.78** ^a^	**0.77** ^a^	0.6^a^
${Entropy}_{y-8}$				**1**	**0.53** ^a^	**-0.37** ^b^	**0.57** ^a^	0.36^b^
${Entropy}_{Angle-1}$					**1**	**-0.78** ^a^	**0.71** ^a^	0.69^a^
${Trend}_{x-3}$						**1**	**-0.73** ^a^	-0.75^a^
$\lambda_{s_{y-8}}$							**1**	0.74^a^
$\lambda_{L_{y-7}}$								1

^a^ Correlation is significant at the 0.01 level.

^b^ Correlation is significant at the 0.05 level.

$\sigma_{r_{y-8}}$ Expresses phase plane portrait in ML direction for trial num#8 (foam + both vibrations).

## 4 Discussion

This study aimed to identify the best performance indicators and sensory conditions to differentiate NSLBP patients from healthy controls, incorporating various surface conditions (rigid or foam), muscle vibration conditions (triceps, multifidus, none, or both), and leveraging multiple linear (LDA) and non-linear (Lyapunov and RQA)) mathematical tools. The findings indicated that there were significant differences in the majority of the parameters used in this experiment (linear parameters, RQA, and Lyapunov components) between the NSLBP and healthy cohorts. The identification of the best indicators and sensory conditions could enable clinicians to distinguish individuals with proprioception impairment. Our results show that using linear discriminant analysis, only two parameters ( $\sigma_{r_{y}}$ , and Entropy ML), as well as the sensory condition of foam surface with vibration on both the ankle and back, are sufficient to separate two cohorts of Healthy and NSLBP individuals. Although the use of foam surface has been reported exclusively as a sensory condition to discriminate the LBP patients from controls (Salavati et al., 2009), our investigation showed that the addition of vibration has a powerful impact confirming the hypothesis (Figure 2). Importantly, this implies that the current results could provide a direction for future studies aiming to investigate a diagnostic framework for people with proprioception disorders. In our previous work (Davoudi et al., 2020) we classified Chronic Non-specific Low Back pain patients (CNLBP) into three subgroups of low, medium and high risk by linear discriminant analysis based on special sensory conditions and IMU sensors. Our current results suggest that Low Back Pain patients could be potentially distinguished from healthy individuals by employing a sensory condition and linear indicators, although future work is required for further validation.

Psychological variables have been shown to impact mobility and postural balance, in addition to LBP (Pincus et al., 2002). One meta-analysis study found a significant, albeit modest, link between negative emotional characteristics (such as anxiety, heightened fear of movement or pain) and more stiff and protective spinal movement (Christe et al., 2021a). The study suggested further investigation of “specific and personalized assessment of psychological variables, pain severity, and spinal motor behavior” (Christe et al., 2021b). Another study Wernli K et al. (2022) demonstrated that each participant had an individual recovery journey from conscious and non-conscious protection to conscious and non-conscious non-protection. Prior to and following cognitive functional therapy intervention for 12 patients with disabling LBP, pre- and post-quantitative measures of movement, posture, psychological factors, pain, and activity limitation integrated well with the findings from qualitative interviews. Targeting psychological variables may thus be crucial for regaining normal posture and should be further explored in similar studies.

Koch et al. (2020) used COM and surface electromyography in NSLBP and healthy individuals during standing on rigid or foam surface with open and occluded eyes. Although the COM value was higher in low back pain patients, which is consistent with further use of the ankle strategy, the COM and EMG parameters did not show any significant differences between low back pain patients and healthy individuals. By contrast, in this study, most indicators revealed significant differences between the NSLBP patients and healthy groups. It seems therefore that that applying vibration on ankle and back muscles had a important impact on biasing the motor control impairment in non-specific low back pain patients, which enabled the use of simple indicators instead of EMG for group distinction. Nogueira et al. (2020) found multiple discriminant variables based on COP data analysis, although their study did not investigate the effects of unstable surface and/or the effect of vibration on motor control impairment in low back pain patients. Posture control changes radically on an unstable surface (foam) or by biasing the proprioception signals (for example by muscle vibration). Ito et al. (2018) showed that changes in ankle or hip strategy proprioceptive signals lead to changes in NSLBP patients at different frequencies. On the other hand, there are multiple studies which could distinguish the LBP patients form healthy individuals based on trunk motion analysis (McDowell et al., 2018). These studies, however, do not provide much insight on associated motor control impairment. In this work, we employed the two main factors (surface and muscle vibration) with major impact on determining the motor control strategy for preserving the body stability. This approach enables us to compare and differentiate the motor control strategy in low back pain patients with that in healthy individuals towards diagnosing proprioceptive impairment and making informed treatment decisions. In contrast with our hypothesis, linear indicators could be of more value in future studies for group discrimination $\sigma_{r_{y}}$ (100%), while the ML direction seems also to play a key role in the distinction of the two cohorts.

There were several noteworthy limitations in this study. Firstly, linear discriminant analysis requires more input data to correctly examine the differences of indicators in both cohorts of low back pain patients and healthy individuals. This study should therefore be expanded to include more healthy and low back pain patients. Secondly, this investigation was conducted only males due to logistics and hence looking at females and examining gender differences is an important future direction. Another limitation of this study is that only linear discriminant analysis was performed. Machine learning and/or non-linear discriminant methods can be utilized for better accuracy and sensitivity.

## 5 Conclusion

The main contribution in this study is the development of a relatively simple methodology leveraging linear and non-linear mathematical tools to investigate the main factors (surface and muscle vibration) impacting motor control strategy towards the quantitative discrimination between NSLBP patients with proprioceptive disorders from healthy individuals. LDA analysis identified two indicators (PPP_ML_ and Entropy_ML_) on a foam surface, with both ankle and back vibration, that could discriminate the two groups successfully. The current study sheds light on the need to provide a standardized quantitative platform to distinguish NSLBP populations from healthy groups and offers a starting point for future studies to help clinicians assess the sensory status of patients towards preventive medicine and precise patient-centric rehabilitation. Further work is needed to increase the size and diversity of the population studied in order to validate the present results while cautiously recommending it for clinical applications.

## Data Availability

The raw data supporting the conclusion of this article will be made available by the authors, without undue reservation.
